# Genomic characterization of eight novel *Bartonella* species from bats and ectoparasites reveals phylogenetic diversity and host adaptation

**DOI:** 10.1371/journal.pntd.0013646

**Published:** 2025-10-23

**Authors:** Wen-Jie Wang, Qiao Gao, Bing-Zhou Guo, Xiao Xiao, Hui-Ju Han

**Affiliations:** 1 School of Public Health, Shandong First Medical University, Jinan, Shandong, China; 2 Microbiological Laboratory, Yantai Center for Disease Control and Prevention, Yantai, Shandong, China; 3 College of Medical Information and Artificial Intelligence, Shandong First Medical University, Jinan, Shandong, China; 4 Hubei Shizhen Laboratory, Lab Animal Research Center, Hubei University of Chinese Medicine, Wuhan, Hubei, China; University of Texas Medical Branch, UNITED STATES OF AMERICA

## Abstract

Bats are recognized as hosts of diverse *Bartonella* species, and *Bartonella* are known agents of human disease. Here, we reported the isolation of 26 *Bartonella* strains belonging to eight distinct species from two bat species (*Myotis laniger* and *M. adversus*) and their ectoparasites (bat flies and bat mites) collected from Chongming County, Hubei Province, China. We obtained whole genome sequences of the bat-borne *Bartonella* spp. with second and third generation sequencing. The genome size of the bat-borne *Bartonella* spp. ranged from 1.39 to 1.98 Mb, with the GC content from 35.30%-38.92%. Phylogenetic analysis revealed that the bat-borne *Bartonella* spp. were divergent from currently known *Bartonella* spp., and the Average Nucleotide Identity (ANI) values were all below 95%, indicating that they were all novel species. Comparative genomic analysis underscored distinct characteristics of bat-borne *Bartonella* spp. in cell motility, replication, recombination, and the biogenesis of the cell wall/membrane/envelope, suggesting that bat-borne *Bartonella* spp. exhibited unique host adaptability. Notably, the virulence factor genes of two bat-borne *Bartonella* spp., strains B23 and 39, were highly analogous to those of the currently known *Bartonella henselae* and *Bartonella quintana*, indicating that they might be pathogenic to humans and animals. Altogether, our findings significantly broaden the diversity of the *Bartonella* genus, and provide new insights into the host specificity and evolutionary relationship of bat-borne *Bartonella* spp..

## Author summary

Bats and their ectoparasites are natural hosts of many bacteria that may cause disease in humans, especially in tropical and subtropical regions. In this study, we discovered eight previously unknown *Bartonella* species from bats and their parasites in China. By analyzing their genomes, we found that these bacteria are genetically distinct and carry features suggesting they have adapted to specific hosts. Some also contain genes similar to those found in known human pathogens, raising concern about their potential to infect humans or animals. This research improves our understanding of bacterial diversity in wildlife and highlights the importance of monitoring lesser-known microbes that may pose future health risks.

## Introduction

*Bartonella* spp. are a group of Gram-negative, facultative intracellular bacteria, which infect and replicate in endothelial cells and erythrocytes [1]. Currently, the genus *Bartonella* comprises 59 confirmed species and 17 provisional species and many *Bartonella* species are associated with human diseases including *Bartonella bacilliformis* (Carrion′s disease), *B. henselae* (cat-scratch disease), and *B. quintana* (trench fever) [2,3]. Moreover, emerging novel *Bartonella* spp. are reported to be responsible for blood-culture-negative endocarditis [4,5]. *Bartonella* have a wide range of mammalian hosts, including rodents, cats, and dogs [6–8]. Bats, belonging to the Order Chiroptera, are important reservoirs of emerging infectious diseases [9]. Globally, a diversity of *Bartonella* spp. are identified in bats and their ectoparasites, highlighting the role of bats as hosts of *Bartonella* species [10,11]. Currently, our understanding of bat-borne *Bartonella* spp. is limited due to the scarcity of whole genome data. Such genomic data are important for comprehensive characterization of their genetic diversity, the identification of key virulence determinants, and the elucidation of evolutionary adaptations underlying host specificity and potential zoonotic transmission. In our previous work, a diversity of novel *Bartonella* spp. in bats and their ectoparasites in China was identified using multi-locus PCR approaches [12,13]. To better understand bat-borne *Bartonella*, we herein aim to isolate *Bartonella* from bats and their ectoparasites, and to obtain the whole genome sequences of bat-borne novel *Bartonella* spp. for phylogenetic analysis and comparative genomic analysis. Our findings will significantly broaden the diversity of the *Bartonella* genus and provide new insights into the host specificity and evolutionary relationships of bat-borne *Bartonella* spp..

## Materials and methods

### Ethics statement

The collection of bats for *Bartonella* isolation was approved by the Ethics Committee of Shandong First Medical University (W202303060236), and all efforts were made to minimize discomfort to bats. The study was conducted in strict accordance with the institutional guidelines.

### Sampling and species identification of bats and their ectoparasites

Bats were sampled from Chongming County, Hubei Province, China, using mist nets placed at the entrances of karst caves during peak activity periods of bats (at sunset and before dawn). Nets were continuously monitored, and captured bats were promptly removed upon capture. Captured bats were placed in cloth bags, euthanized by inhalation of ethyl ether in the field with efforts to minimize animal discomfort, and transported on ice to the laboratory for further analysis. Bat flies were collected from the fur of bats, and bat mites were obtained from their wing membranes. Bat spleens were collected via necropsy and specimens were stored at −80°C until use. Bats and their ectoparasites were identified by PCR amplification and subsequent sequencing of the cytochrome B (*cytB*) and cytochrome oxidase subunit I (*COI*) genes, respectively [13].

### Bartonella isolation and molecular identification

As an ongoing program aimed at identifying pathogens in bats, the spleen was used for *Bartonella* isolation in this study, which typically harbors higher bacterial loads than blood, facilitating the detection and isolation of *Bartonella* [14,15]. Bat spleen samples were ground with 200 μL Schneider’s insect medium (Sigma-Aldrich, Germany) supplemented with 10% fetal bovine serum (BBI, China) and 2% amphotericin B (Solarbio, China). Bat ectoparasites were washed with 75% alcohol and then washed twice with PBS before being homogenized using a handheld electric homogenizer. An aliquot of 100 μL homogenate was inoculated onto a Columbia blood agar plate (Oxoid, UK) supplemented with 10% defibrinated sheep blood (Solarbio, China). The plates were cultured in a humidified incubator at 37°C, and 5% CO_2_, and were checked daily for *Bartonella* colonies for a month. Each suspected *Bartonella* isolate was obtained from a single colony, subcultured through three passages, and identified by PCR amplification and sequencing of the 16S rRNA, *gltA*, *rpoB*, and *ftsZ* genes with primers as described previously [13,16]. Based on the primer schemes employed in previous studies, modifications were made in this study. The primers used for 16S rRNA, *gltA*, *rpoB*, and *ftsZ* are provided in S6 Table. Maximum likelihood phylogenetic trees were constructed for the 16S rRNA, *gltA*, *rpoB*, and *ftsZ* genes using IQ-TREE with 1,000 bootstrap replicates [17]. The best-fitting nucleotide substitution model for each gene was selected based on the Bayesian Information Criterion (BIC), resulting in K2P+I + G4, TIM3 + F + R3, TN + F + R3, and HKY + F + R2 for the 16S rRNA, *gltA*, *rpoB*, and *ftsZ* genes, respectively.

### Whole-genome sequencing and phylogenetic analysis

Isolated *Bartonella* spp. were scraped off the Columbia blood agar plates, and genomic DNA was extracted with NucleoBond HMW DNA Kit (Takara, Japan). Genomic DNA was subjected to both short- and long-read sequencing. For short-read sequencing, DNA was randomly fragmented to approximately 300 bp to construct paired-end libraries, which were sequenced on the MGI DNBSEQ-T7 platform using a 150 bp paired-end strategy. For long-read sequencing, DNA was sheared with g-TUBEs (Covaris, USA), size-selected and purified with AMPure PB beads, and libraries were prepared using the SMRTbell Express Template Prep Kit 2.0 (Pacific Biosciences, USA). The polymerase–primer complexes were generated with the Sequel II Binding Kit 2.0, and sequencing was performed on the PacBio Sequel II platform according to the manufacturer’s instructions. Fastp was used to remove adapter sequences and low-quality sequences from the second-generation data, and Fastplong was used for quality control on the third-generation sequencing data [18,19]. The genome was assembled using Spades [20], and the completeness and accuracy of genome assembly were assessed using BUSCO and CheckM, respectively [21,22]. Prokka was used for protein annotation [23]. Orthofinder was employed to identify single-copy orthologous proteins, each orthologous gene was aligned using Mafft with the FFT-NS-2 algorithm and the same parameters across all genes [24,25]. The resulting alignments were concatenated into a supermatrix, which was then used to construct a whole-genome-based phylogenetic tree with IQ-TREE with 1,000 bootstrap replicates. The best nucleotide substitution model Q.bird+R + F was selected based on the Bayesian Information Criterion (BIC), and was visualized with iTOL [17,26]. FastANI was used to compute the overall Average Nucleotide Identity (ANI), with 95% as the cutoff to define species boundaries [27,28]. DNA-DNA hybridization (DDH) values were calculated using an online tool (ggdc.dsmz.de), and a threshold of 70% was applied for species delineation [29,30].

### Genome annotation and pangenome analysis

Genome sequences of reference *Bartonella* spp. were downloaded from the NCBI RefSeq (https://www.ncbi.nlm.nih.gov/refseq/) [31] (S3 Table). Functional characterization of the *Bartonella* genomes was conducted using the EggNOG-mapper [32], and virulence factor annotation was conducted using Diamond against the VFDB database [33]. Core genes and unique genes were identified through Roary analysis [34].

### Positive selection analysis and genome comparison

After the core gene sequences were obtained, they were translated into coding sequences (CDs) using the Gffread software [35]. The aligned protein sequences, together with their corresponding CDs were processed using PAL2NAL to produce codon-based alignments suitable for selection pressure analysis [36]. The sequence alignments generated by PAL2NAL were used to construct a phylogenetic tree using IQ-TREE [17]. Based on the phylogenetic tree and the aligned sequence, selection pressure analysis was performed using the aBSREL model in HyPhy to identify positively selected genes among the core genes [37]. Positively selected genes were extracted from the core genes using Python scripting. Functional annotation of these positively selected genes was conducted employing EggNOG-mapper [32].

For strains B30 and B17, which were isolated from the same host, genome alignment was performed using Bowtie2 [38]. The alignment results were indexed and single nucleotide polymorphism (SNP) regions were extracted using FreeBayes [39]. The SNP regions were annotated with snpEff [40]. After the identification of SNP sites with significant impact, genes containing these loci were functionally annotated using EggNOG-mapper [32]. The versions and parameter settings of all software used in this study are provided in S5 Table.

## Results

### Sampling and species identification of bats and their ectoparasites

A total of 28 bats, including *Myotis laniger* and *Myotis adversus*, were examined, along with 49 ectoparasites, comprising 34 *Penicillidia monoceros*, five *Nycteribia formosana* (processed as ten pooled samples), and ten *Spinturnix* sp. (processed as 23 pooled samples). Among the bat spleen samples, eight of 28 (28.6%) were positive for *Bartonella*, including six positives from *M. laniger* and two from *M. adversus*. For ectoparasites, nine of 34 *P. monoceros* (26.5%) were positive, eight of ten pooled *N. formosana* samples (80.0%) were positive, and one of 23 pooled *Spinturnix* sp. samples (4.3%) was positive. Percentages indicate the proportion of positive samples among those tested in this study.

### *Bartonella* isolation and molecular identification

**Fig 1:**
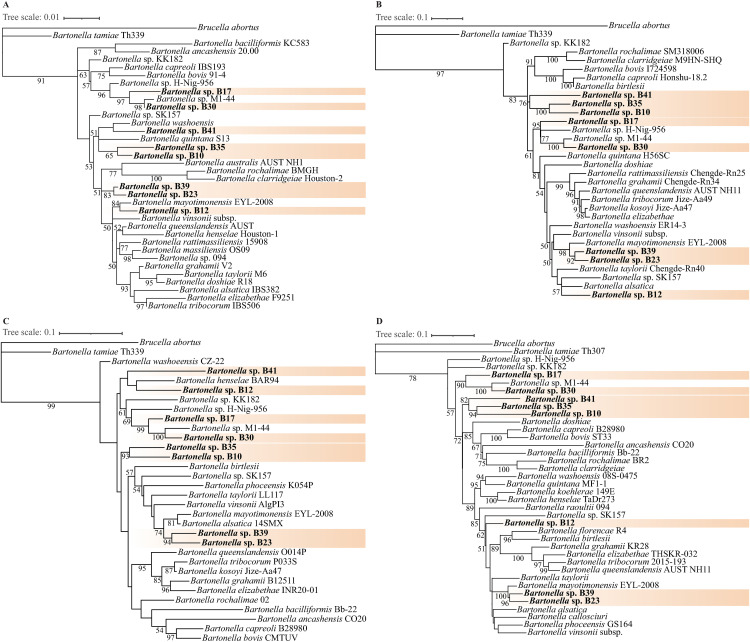
Phylogenetic analysis of the eight bat-borne novel *Bartonella* spp. of this study based on the gene of 16S rRNA (A), *ftsZ* (B), *gltA* (C) and *rpoB* (D), respectively. The trees were constructed using the best nucleotide substitution model selected based on the Bayesian Information Criterion (BIC), with K2P+I + G4, TIM3 + F + R3, TN + F + R3, and HKY + F + R2 for 16S rRNA, *gltA*, *rpoB*, and *ftsZ*, respectively. The bootstrap values were 1,000. Strains identified in the present study are shown in bold font, with bootstrap values ≥50 indicated at the nodes. The branch length of the outgroup (*Brucella abortus*) was reduced by 30% for visualization purposes.

A total of 26 strains of *Bartonella* were isolated from bat spleens and bat ectoparasites, and phylogenetic analysis based on the genes of 16S rRNA, *gltA*, *rpoB*, and *ftsZ* indicated that these isolates represent eight potential novel *Bartonella* species, which are provisionally designated in this study as *Bartonella* sp. B10, *Bartonella* sp. B12, *Bartonella* sp. B17, *Bartonella* sp. B23, *Bartonella* sp. B30, *Bartonella* sp. B35, *Bartonella* sp. B39, and *Bartonella* sp. B41 (Table 1, Fig 1). All gene sequences and whole-genome data have been deposited in GenBank and accession numbers have been obtained (S3 Table).

**Table 1:** Summary of the bat-borne *Bartonella* isolated in this study.

*Bartonella* strains	*Bartonella* isolates	Host type	Host species
B10	B-10	Bat mite	*Spinturnix* sp.
B30	B-30	Bat fly	*Penicillidia monoceros*
B35	B-35	Bat	*Myotis laniger*
B39	B-39	Bat	*Myotis adversus*
B41	B-41	Bat	*Myotis laniger*
B23	B-23	Bat fly	*Nycteribia formosana*
	B-25	Bat fly	*Nycteribia formosana*
	B-28	Bat	*Myotis laniger*
B12	B-12	Bat fly	*Nycteribia formosana*
	B-14	Bat fly	*Nycteribia formosana*
	B-24	Bat fly	*Nycteribia formosana*
	B-26	Bat fly	*Nycteribia formosana*
	B-31	Bat fly	*Nycteribia formosana*
	B-32	Bat fly	*Nycteribia formosana*
	B-37	Bat	*Myotis laniger*
B17	B-15	Bat fly	*Penicillidia monoceros*
	B-16	Bat fly	*Penicillidia monoceros*
	B-17	Bat fly	*Penicillidia monoceros*
	B-18	Bat fly	*Penicillidia monoceros*
	B-19	Bat fly	*Penicillidia monoceros*
	B-20	Bat fly	*Penicillidia monoceros*
	B-21	Bat fly	*Penicillidia monoceros*
	B-22	Bat fly	*Penicillidia monoceros*
	B-36	Bat	*Myotis laniger*
	B-38	Bat	*Myotis laniger*
	B-40	Bat	*Myotis adversus*

### Genomic characteristics and phylogenetic analysis

**Fig 2:**
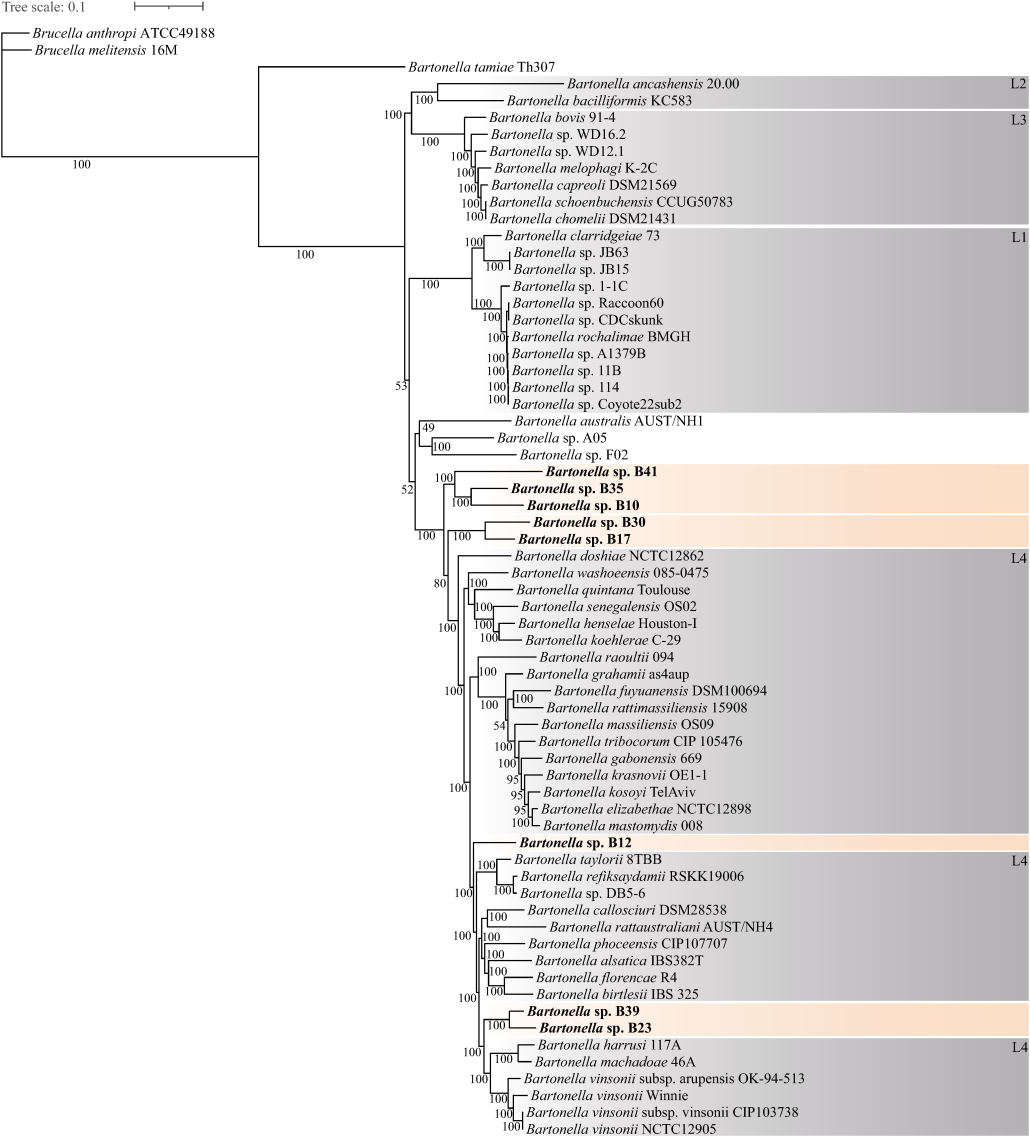
Whole-genome-based phylogenetic analysis of *Bartonella* with 545 single-copy orthologous genes using the ML method with a bootstrap value of 1,000. Strains identified in the present study are shown in bold font, with bootstrap values ≥50 indicated at the nodes.

The eight bat-borne *Bartonella* spp. isolated in this study underwent second and third-generation sequencing. Assembly completeness, estimated from the presence of lineage-specific single-copy marker genes, ranged from 94.0% to 100%, with contamination levels below 5.39%. The genome size of the bat-borne *Bartonella* spp. ranged from 1.39 to 1.98 Mb, and the GC content ranged from 35.30% to 38.92% (Table 2). Whole genome-based phylogenetic analysis showed that the bat-borne *Bartonella* spp. formed multiple well-supported monophyletic clades (Fig 2), highlighting their distinct evolutionary lineages. Strains B10, B35, and B41 clustered into a stable branch with full bootstrap support (100%), while strains B17 and B30 also formed a strongly supported monophyletic clade (100% bootstrap support). These two distinct lineages represent ancestral branches to the L4 *Bartonella* clade. Similarly, strains B23 and B39 consistently grouped together (100% bootstrap support) and were positioned adjacent to a clade containing *B. machadoe*, *B. harrusi*, and multiple subspecies of *B. vinsonii*. In addition, Strain B12 formed a completely distinct branch within the *Bartonella* lineage L4, without clustering with any of the other strains identified in our study. 

**Table 2:** Genomic characteristics of the eight bat-borne novel *Bartonella* spp. of this study.

Bartonella strains	Genbank accession number	Contig	Total length (bp)	GC content (%)	Completeness (%)	CDs	tRNA genes	rRNA genes
B-10	CP163534	1	1,505,008	36.68	97.75	1,172	42	6
B-12	CP163535	1	1,568,347	38.41	94.71	1,282	38	3
B-17	CP163536	1	1,754,311	37.30	99.10	1,473	42	6
B-23	CP163537	1	1,727,590	37.96	98.97	1,358	42	6
B-30	CP163538	1	1,644,566	36.47	98.97	1,308	42	6
B-35	CP163539	1	1,542,896	35.30	99.45	1,198	42	6
B-39	CP163540	1	1,984,756	38.92	100.00	1,652	42	6
B-41	CP163541	1	1,387,997	37.10	97.55	1,142	42	6

The average nucleotide identity (ANI) values of the bat-borne novel *Bartonella* spp. were all < 95% (79.76% -91.15%), dDDH values were all < 70% (21.8% -29.5%), confirming that they were distinct *Bartonella* spp. (S1 Table). The ANI values among the bat-borne *Bartonella* spp. and their closely related reference *Bartonella* spp. ranged from 77.55% to 86.21%, all below the 95% threshold, and all dDDH values were all < 70% (Tables 3 and S1), indicating that they were novel *Bartonella* spp.. 

**Table 3:** ANI and dDDH values calculated between the eight bat-borne novel *Bartonella* spp. of this study and currently known closely related *Bartonella* spp..

	B10		B12		B17		B23		B30		B35		B39		B41	
A	B	A	B	A	B	A	B	A	B	A	B	A	B	A	B
*B. australis*	78.2	20.2	78.8	21.0	78.5	20.5	78.3	20.1	78.1	19.9	78.6	20.9	78.5	20.7	77.9	19.8
*B. bacilliformis*	79.3	21.1	79.4	21.6	79.6	21.6	79.2	21.5	79.3	21.0	79.8	21.8	79.2	21.5	78.7	20.6
*B. birtlesii*	80.9	23.0	84.3	27.5	81.2	23.6	83.4	26.2	80.5	22.8	81.6	24.0	84.3	27.5	79.8	22.1
*B. doshiae*	80.7	23.4	83.1	26.2	81.5	24.0	82.6	25.2	80.3	22.9	81.8	24.4	83.6	26.7	79.9	22.3
*B. grahamii*	80.4	23.1	83.5	27.0	80.9	23.5	82.7	25.8	80.1	22.9	81.3	24.2	83.9	27.2	79.5	22.0
*B. henselae*	81.1	23.7	84.6	27.7	82.1	24.4	83.4	26.5	80.8	23.4	82.3	24.7	84.4	27.3	79.9	22.6
*B. quintana*	81.5	24.1	85.1	28.2	82.2	24.9	83.8	26.7	81.2	23.6	82.6	25.3	84.6	27.9	80.3	22.9
*B. refiksaydamii*	81.0	23.6	85.9	29.0	81.7	24.1	84.2	27.3	80.6	23.1	82.1	24.5	85.3	28.5	80.0	22.5
*B. tamiae*	82.4	20.4	82.4	21.6	79.3	20.4	79.3	21.8	79.4	19.9	80.4	19.7	79.7	20.4	78.4	20.1
*B. taylorii*	81.4	23.8	86.2	30.1	81.9	24.4	84.5	27.8	80.9	23.2	82.3	24.9	85.7	29.3	80.2	22.8
*B. vinsonii*	80.9	23.5	85.0	28.4	81.5	24.0	84.3	27.6	80.4	23.1	81.7	24.5	85.4	28.9	79.8	22.5

Column A and B represented ANI values and dDDH values, respective.

### Genome annotation and pangenome analysis

**Fig 3:**
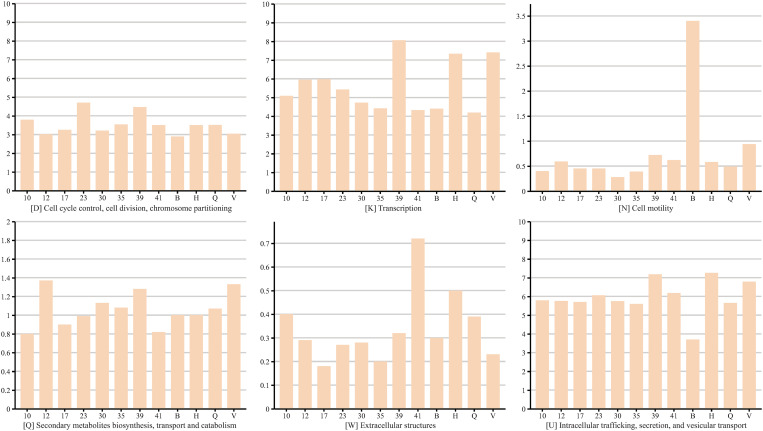
Statistics of COG functional annotation categories for the eight bat-borne novel *Bartonella* spp. of this study and four representative pathogenic *Bartonella* strains. The y-axis represents the proportion of annotated genes assigned to each functional category in the *Bartonella* genomes, while the x-axis denotes different *Bartonella* species, where B indicates *B. bacilliformis* KC583, H indicates *B. henselae* Houston-I, Q indicates *B. quintana* Toulouse, and V indicates *B. vinsonii* NCTC12905.

For comparative purposes, four pathogenic *Bartonella* species with representative pathogenic relevance and high-quality genomes (*B. bacilliformis*, *B. henselae*, *B. quintana*, and *B. vinsonii*) were included in the analysis. Gene annotation with EggNOG-mapper for the eight bat-borne novel *Bartonella* spp. of this study and four pathogenic *Bartonella* spp. showed that the top four most abundant functional categories, in terms of proportion of annotated genes, were J (translation, ribosomal structure and biogenesis), E (amino acid transport and metabolism), L (replication, recombination, and repair), and M (cell wall/membrane/envelope biogenesis) (Figs 3 and S1, S5 Tables). The relative proportion of genes in functional category N (cell motility) in the bat-associated novel *Bartonella* spp. was significantly lower than that of *B. bacilliformis*. Moreover, a markedly higher proportion of genes in functional category K (transcription) was observed in strain B39 compared to the other seven *Bartonella* spp., as well as *B. bacilliformis* and *B. henselae* (Figs 3 and S1). 

**Fig 4:**
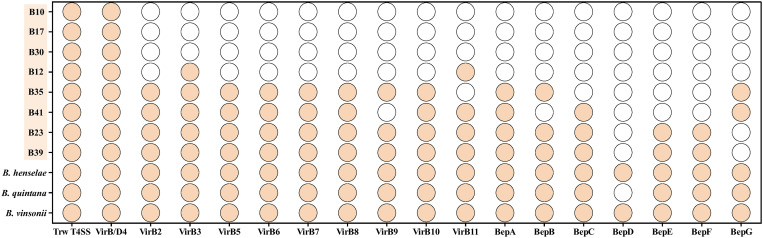
Virulence factors comparison of the eight bat-borne novel *Bartonella* spp. of this study and 3 representative pathogenic *Bartonella* spp.. Solid circles indicate the presence of the virulence factor, while empty circles represent the absence of the virulence factor.

All eight bat-borne novel *Bartonella* spp. isolated in this study contained Type IV secretion system (T4SS) and VirB/D4 (Fig 4). Strains B23 and B39, which clustered together in the phylogenetic tree, were observed to exhibit a similar distribution of virulence factors with the pathogenic *B. henselae*, *B. quintana*, and *B. vinsonii* (Fig 4), consistent with the virulence factor profile characteristic of lineage L4, thereby indicating a closely related evolutionary history and pathogenic potential [41]. 

The core genes, accessory genes, and specific genes of the bat-borne novel *Bartonella* spp. were identified with pan-genomic analysis (S2 Table). Core functional categories, such as J (Translation, ribosomal structure and biogenesis), C (Energy production and conversion), and K (Transcription), were consistently represented by high gene counts across all species (S2 Table).

### Positive selection analysis and genome comparison

Positive selection branch detection was performed with 1,859 core genes of the eight bat-borne novel *Bartonella* spp.. When considering only ω values >1, an average of 74 positively selected genes were found in the core genes of the bat-borne novel *Bartonella* spp., ranging from a minimum of 42 in strain B10 to a maximum of 128 in strain B12. Under the condition of ω value >1 and *P* value <0.05, fewer genes were identified (e.g., 10 in strain B10 and 2 in strains B12 and B41), whereas no genes met this threshold in strain B23 (S4 Table). While some core genes may experience episodic positive selection, the majority are under strong purifying selection. It should be noted that after stringent multiple testing correction, none of the core genes reached statistically significant levels, highlighting the conservative nature of these essential genes.

**Fig 5:**
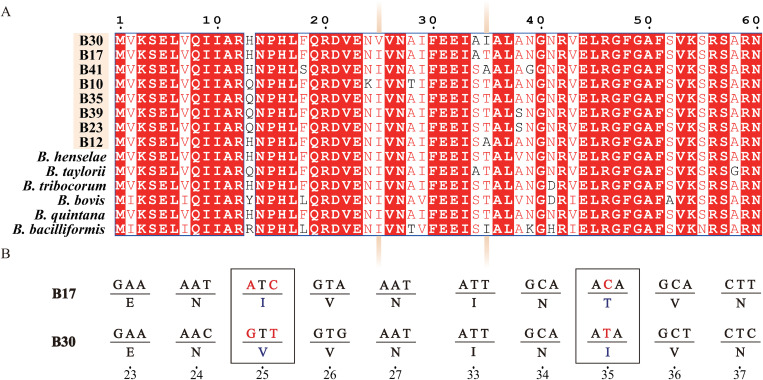
Comparison and SNP analysis of the *himD* gene of Strains B17 and B30. **(A)** Amino acid sequence alignments of HimD in strain B30 with 13 other *Bartonella* HimD. **(B)** Detailed nucleotide alignments of the *himD* gene in strains B17 and B30 showing the codon changes responsible for amino acid substitutions at positions 25 and 35.

In this study, the genome sequences of strains B30 and B17, which were isolated from the same host, were compared to identify significant SNP sites. Significant SNP sites in the *himD* gene were identified through whole-genome comparison (Fig 5B), showing significant genetic differences between the two *Bartonella* spp.. The HimD is one of the two subunits of the integration host factor [42], a specific DNA-binding protein that plays an important role in gene recombination, transcription, and translation control [43,44]. Compared with 13 reference *Bartonella* spp., the *himD* gene in strain B30 was found to have undergone nonsynonymous substitutions at the 25th and 35th amino acid positions (Fig 5A). Compared to strain B17, two nonsynonymous SNPs were identified in the HimD gene of strain B30, resulting in amino acid substitutions at positions 25 (Isoleucine → Valine) and 35 (Threonine → Isoleucine). Furthermore, signals of positive selection were detected in the *himD* gene of strain B30 (Fig 5B).

## Discussion

By combining isolation and whole-genome sequencing, the eight novel bat-borne *Bartonella* spp. were characterized in this study. The sequencing results indicated that the genome length, GC content, and assembly completeness of the new *Bartonella* species were within the typical ranges observed for the genus (S3 Table). Phylogenetic analyses based on the 16S rRNA, *ftsZ*, *gltA*, and *rpoB* genes consistently supported the placement of the bat-associated *Bartonella* strains into multiple, well-supported and independent clades, which were in full agreement with the phylogeny reconstructed from concatenated single-copy core genes. Based on the whole-genome-based phylogenetic placement, strain B12 was inferred to represent an independent lineage likely belonging to the broader L4 clade. However, the distribution of virulence factors in strain B12 differed from that of the L4 lineage. Notably, strains B23 and B39 clustered closely with Candidatus *Bartonella mayotimonensis* in the phylogenetic trees constructed from *gltA* and *rpoB* sequences, a species originally identified from a human endocarditis patient and later reported in bats [45,46]. This finding underscores the potential pathogenic relevance of bat-associated *Bartonella*. In the whole-genome-based phylogeny, major internal nodes received full bootstrap support, with strains B23 and B39 consistently forming a strongly supported pair (100% bootstrap support) adjacent to the L4 clade containing *B. machadoe*, *B. harrusi*, and the complex of *B. vinsonii* subspecies. These results highlight that the newly described bat-associated *Bartonella* strains expand the phylogenetic diversity of the genus and reveal evolutionary links to clades associated with other mammalian hosts, including rodents, marsupials, and carnivores.

*Bartonella henselae* and *B. quintana* are two well-known pathogenic *Bartonella* spp. [3,47]. Among the bat-borne *Bartonella* species identified in our study, strains B23 and B39 exhibited similar distribution of the virulence factors with *B. henselae* and *B. quintana*. The type IV secretion system (T4SS), comprising VirB and associated *Bartonella* effector proteins (Beps), plays a central role in the pathogenesis of *Bartonella* species by delivering effector proteins into host cells [41,48]. These effectors modulate host cell signalling, inhibit immune responses, enhance bacterial uptake and intracellular survival, and facilitate other pathogenic processes [41,49]. Both strains B23 and B39 were found to possess complete sets of T4SS-related genes, and together with their phylogenetic positions within the genus *Bartonella*, this indicates their potential to infect mammalian hosts and exert pathogenic effects.

Although previous studies have revealed a high diversity of *Bartonella* species in bats and bat ectoparasites based on short gene fragments such as *gltA*, *rpoB*, or *ftsZ* [13,50,51], comprehensive whole-genome data of bat-associated *Bartonella* spp. remain scarce. In this study, a combination of the second and third-generation sequencing methods was used to obtain whole-genome sequences of the novel bat-borne *Bartonella* spp.. Analysis of COG functional annotations among the bat-associated *Bartonella* species core genes revealed a highly conserved distribution pattern across most categories. Compared to other strains, a higher proportion of genes in functional category K (transcription) was shown by 311-B39, suggesting a potentially richer and more active gene expression during transcription. [48,52]. Additionally, several important SNP sites were identified, including a mutation site in the *himD* gene, and positive selection analysis revealed the *himD* gene as an episodically positively selected gene in strain B30. The HimD gene encodes the integration host factor (IHF) subunit, and it was demonstrated that IHF assisted in regulating chromosome function in bacteria by influencing the morphology and organisation of DNA [53]. The transcription of type III secretion system (T3SS) genes and the replication of the pYGK plasmid were also shown to require the involvement of IHF [54,55]. Moreover, the integration host factor has been demonstrated to be essential for controlling virulence gene expression in several bacterial species, including *Salmonella*, *Shigella,* and *Brucella* [56–59]. Positive selection of the IHFβ subunit in strain B30 may be related to its adaptation to the host environment. Under immune or other environmental pressures, changes in the IHFβ subunit may aid strain B30 in evading host defense mechanisms, enhancing its survival and colonization within the host [60]. Strain B30, which was detected exclusively in *Penicillidia monoceros* samples, may reflect adaptive pressures associated with its specialized evolutionary adaptation to bat ectoparasites. In contrast, strain B17 was detected in both ectoparasites and two bat species, whereas strains B39 and B41 were detected only in bat species. Future researches are needed to explore the impact of genetic variation on the function of IHFβ, as well as its effect on host specificity.

In conclusion, with the availability of the whole-genome sequence of bat-borne *Bartonella* spp., our study significantly enhanced the understanding of *Bartonella* spp. diversity and offered novel perspectives on the pathogenicity, virulence mechanisms, and evolutionary adaptations of bat-borne *Bartonella* spp..

## Conclusions

Through isolation and whole-genome sequencing, eight novel bat-associated *Bartonella* species were identified. Their distinct phylogenetic positions, virulence gene profiles, and functional genomic features revealed previously unrecognized diversity within the genus. Strains B23 and B39 were found to carry a complete set of T4SS genes, indicating potential pathogenicity. In strain B30, a mutation site in the *himD* gene suggested possible adaptation to a specific host environment. Our findings provided important genomic insights into the evolution, host specificity, and virulence potential of bat-associated *Bartonella* species, highlighting the necessity for continued surveillance and functional investigations.

## Supporting information

S1 Fig Supplementary figure of the COG database annotation results. (TIF)

S1 Table ANI values and dDDH values of the eight bat-borne novel *Bartonella* spp. of this study. (PDF)

S2 Table Statistical results of pan-genome analysis and core gene functional annotation of the eight bat-borne novel *Bartonella* spp. (PDF)

S3 Table Genomic and gene sequence information (16S rRNA, *gltA*, *ftsZ*, *rpoB*) with accession numbers. (PDF)

S4 Table Episodic positive selection analysis of core genes in bat-borne *Bartonella* was conducted. (PDF)

S5 Table Functional categories of COG protein function annotations and the software versions with parameter settings used in this study. (PDF)

S6 Table Primers used for PCR in this study. (PDF)

S1 File Raw PCR positivity data and associated metadata for *Bartonella* detection in bats and their ectoparasites. This dataset provides sample-level PCR positivity data for bats and their ectoparasites. The table includes the following variables: Sample ID, Animal ID, Latitude, Longitude, Sample collection method, Sample material, Host identification, Detection target, Detection method, Detection outcome, and Species identification. PCR results are represented as “1” for positive and “0” for negative detections. Missing values are indicated by blank cells.
(XLSX)
