# Supplementary figures and images for: Genomic characterization of eight novel Bartonella species from bats and ectoparasites reveals phylogenetic diversity and host adaptation
Source: PLoS Negl Trop Dis. 2025 Oct 23;19(10):e0013646. doi: 10.1371/journal.pntd.0013646 (PMC12574864; doi:10.1371/journal.pntd.0013646)

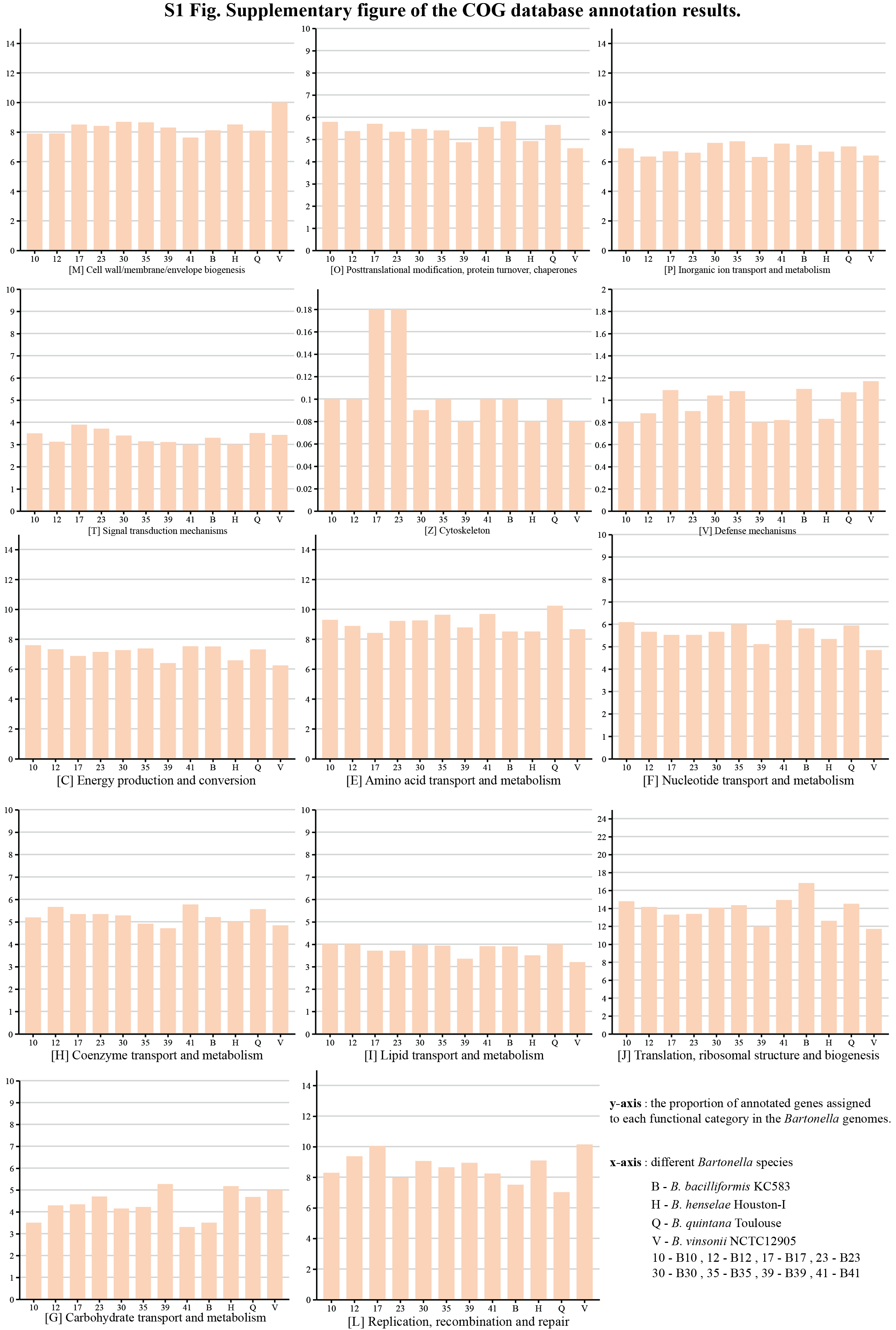

Supplement: S1 Fig — (TIF) [file pntd.0013646.s001.tif]
