# Supplementary material for: Genomic characterization of eight novel Bartonella species from bats and ectoparasites reveals phylogenetic diversity and host adaptation
Source: PLoS Negl Trop Dis. 2025 Oct 23;19(10):e0013646. doi: 10.1371/journal.pntd.0013646 (PMC12574864; doi:10.1371/journal.pntd.0013646)
Supplement: S1 Table — (PDF) [file pntd.0013646.s002.pdf]

S1 Table. ANI values and dDDH values of the eight bat-borne novel *Bartonella* spp. of this study.

	B10		B12		B17		B23		B30		B35		B39		B41	
	A	B	A	B	A	B	A	B	A	B	A	B	A	B	A	B
B10	100.0	100.0	81.5	23.9	81.3	23.5	80.9	23.4	80.6	22.5	84.8	27.8	81.3	24.1	80.6	23.0
B12	81.5	23.9	100.0	100.0	83.1	25.6	84.6	27.7	81.6	24.0	82.9	25.0	85.5	28.5	80.3	22.7
B17	81.3	23.5	83.1	25.6	100.0	100.0	81.8	24.1	86.1	29.5	82.5	24.4	81.9	24.3	80.0	22.6
B23	80.9	23.4	84.6	27.7	81.8	24.1	100.0	100.0	80.6	23.0	81.6	24.3	91.2	41.9	79.8	22.2
B30	80.6	22.5	81.6	24.0	86.1	29.5	80.6	23.0	100.0	100.0	81.3	23.3	80.8	23.0	79.7	21.8
B35	84.8	27.8	82.9	25.0	82.5	24.4	81.6	24.3	81.3	23.3	100.0	100.0	82.0	24.6	81.3	23.9
B39	81.3	24.1	85.5	28.5	81.9	24.3	91.2	41.9	80.8	23.0	82.0	24.6	100.0	100.0	80.1	22.6
B41	80.6	23.0	80.3	22.7	80.0	22.6	79.8	22.2	79.7	21.8	81.3	23.9	80.1	22.6	100.0	100.0

Column A and B represented ANI values and dDDH values, respectivel

S1 Table. ANI and dDDH values of the eight novel *Bartonella* spp. and other *Bartonella* species.

	B10		B12		B17		B23		B30		B35		B39		B41	
	A	B	A	B	A	B	A	B	A	B	A	B	A	B	A	B
B. ancashensis	77.8	19.9	77.9	19.6	77.9	19.8	77.7	19.5	77.9	19.9	78.1	19.9	77.8	19.8	77.6	19.9
B. australis	78.2	20.2	78.8	21.0	78.5	20.5	78.3	20.1	78.1	19.9	78.6	20.9	78.5	20.7	77.9	19.8
B. bovis	79.7	21.9	80.2	22.1	79.6	21.9	79.8	21.9	79.2	21.2	80.3	22.4	80.0	22.1	78.9	20.9
B. callosciuri	80.7	23.5	85.4	28.4	81.4	24.1	83.8	26.6	80.5	23.3	81.9	24.3	84.9	27.9	79.7	22.2
B. capreoli	79.5	21.7	80.2	22.7	79.9	22.3	79.7	22.3	79.4	21.4	80.0	22.5	79.8	22.6	79.3	21.0
B. chomelii	79.6	21.8	80.1	22.0	79.9	22.0	79.6	22.1	79.5	21.4	80.2	22.5	79.8	22.2	79.1	20.9
B. clarridgeiae	79.3	21.3	79.6	21.8	79.5	21.8	79.4	21.3	79.3	21.3	80.2	22.4	79.6	22.0	78.8	20.6
B. elizabethae	79.7	22.5	82.6	26.0	80.3	23.0	81.6	24.7	79.7	22.5	80.7	23.5	82.2	25.5	78.8	21.3
B. florencae	80.2	22.8	83.9	26.8	80.8	23.1	82.9	25.9	80.1	22.6	81.2	23.8	83.9	26.7	79.5	22.0
B. fuyuanensis	79.6	22.2	82.4	25.3	80.3	22.8	81.3	24.3	79.5	22.0	80.6	23.1	81.9	25.0	79.1	21.5
B. gabonensis	80.1	22.3	82.9	25.8	80.5	23.1	81.6	24.7	79.7	22.3	80.6	23.3	82.3	25.4	79.1	21.5
B. harrusi	80.2	22.7	83.5	26.8	80.7	23.3	83.8	26.9	80.1	22.7	81.1	23.8	84.6	27.9	79.3	21.9
B. koehlerae	80.9	23.5	84.5	27.4	81.6	24.2	83.3	26.2	80.9	23.2	81.9	24.5	83.9	27.1	80.0	22.2

B. kosoyi	79.9	22.7	82.7	25.8	80.3	23	81.6	24.6	79.7	22.4	80.7	23.6	82.3	25.6	79.0	21.6
B. krasnovii	79.9	22.5	82.6	25.8	80.4	23.1	81.4	24.5	79.8	22.5	80.5	23.4	82.1	25.4	79.3	21.6
B. machadoae	80.2	22.8	83.5	26.5	80.7	23.3	83.7	27.1	79.9	22.7	81.1	23.7	84.5	27.6	79.4	21.8
B. massiliensis	79.8	22.3	82.4	25.5	80.4	22.9	81.4	24.4	79.6	22.5	80.5	23.1	82.0	25.2	78.9	21.4
B. mastomydis	79.7	22.4	82.6	25.9	80.3	23.0	81.5	24.7	79.8	22.3	80.5	23.4	82.0	25.4	78.9	21.4
B. melophagi	79.6	21.6	79.9	22.1	79.8	22.0	79.5	22.0	79.4	21.2	80.2	22.3	79.8	22.3	78.9	20.9
B. phoceensis	80.3	23.1	84.8	27.5	80.9	23.4	84.2	27.1	80.4	22.7	81.5	24.1	85.1	28.2	79.5	22.1
B. quintana	81.5	24.1	85.1	28.2	82.2	24.9	83.8	26.7	81.2	23.6	82.6	25.3	84.6	27.9	80.3	22.9
B. raoultii	80.4	22.6	82.4	25.2	80.7	23.2	81.5	24.5	80.1	22.5	81.2	23.5	82.3	25.2	79.6	21.8
B. rattaustraliani	79.9	22.7	82.8	26.0	80.4	23.1	81.6	24.9	79.7	22.2	80.6	23.4	82.4	25.7	79.2	21.4
B. rattimassiliensis	80.0	22.5	82.4	25.6	80.5	23.0	81.3	24.6	79.7	22.1	80.9	23.3	82.3	25.2	79.1	21.6
B. rochalimae	79.2	21.1	79.3	21.4	79.2	21.5	79.1	21.1	79.3	20.8	79.7	22..0	79.3	21.7	78.5	20.9
B. schoenbuchensis	79.7	21.8	80.1	22.1	79.9	22.1	79.7	22.1	79.3	21.5	80.1	22.4	79.9	22.2	79.1	20.9
B. senegalensis	80.8	23.3	83.8	27.0	81.3	24.0	82.6	25.9	80.4	23.1	81.5	24.4	83.5	26.6	79.8	22.0
B. sp 1-1C	79.3	21.2	79.3	21.5	78.9	21.6	79.1	21.3	78.9	20.9	79.7	21.9	79.4	21.7	78.5	20.7
B. sp 11B	79.2	21.2	79.2	21.5	79.3	21.7	79.3	21.3	79.2	20.9	79.7	22.0	79.5	21.9	78.6	21.1

B. sp 114	79.2	21.2	79.3	21.5	79.2	21.6	79.2	21.3	79.3	20.9	79.7	22.0	79.4	21.8	78.5	21.0
B. sp A1379B	79.3	21.1	79.3	21.5	79.3	21.6	79.3	21.2	79.3	20.9	79.5	22.0	79.4	21.8	78.5	20.9
B. sp CDC	79.1	21.1	79.2	21.5	79.3	21.5	79.1	21.2	79.2	20.9	79.6	22.0	79.5	21.9	78.6	21.0
B. sp Coyote22	79.1	21.2	79.3	21.5	79.4	21.6	79.3	21.3	79.2	20.9	79.7	22.0	79.3	21.9	78.5	21.0
B. sp DB5-6	81.1	23.7	85.6	29.1	81.6	24.2	83.9	27.3	80.6	23.2	82.1	24.7	85.1	28.6	80.1	22.8
B. sp JB15	79.4	21.2	79.6	21.8	79.5	21.5	79.3	21.4	79.1	21.0	79.9	22.2	79.3	21.7	78.7	20.7
B. sp JB63	79.4	21.2	79.6	21.8	79.6	21.5	79.3	21.4	79.2	21.0	79.9	22.2	79.4	21.7	78.6	20.6
B. sp Raccoon60	79.2	21.1	79.2	21.5	79.1	21.6	79.2	21.2	79.2	21.0	79.6	22.0	79.4	21.9	78.5	20.9
B. sp WD121	79.8	21.8	80.0	22.1	79.8	22.0	79.5	22.0	79.3	21.6	80.3	22.6	79.8	22.2	79.3	21.1
B. sp WD162	79.6	21.9	80.1	22.0	79.7	22.0	79.5	21.8	79.4	21.4	80.5	22.4	79.7	22.2	78.9	20.9
B. tribocorum	79.7	22.5	82.6	25.9	80.4	22.7	81.6	24.7	79.7	22.3	80.7	23.4	82.6	25.8	78.9	21.7
B. vinsonii OK-94-513	80.8	23.6	85.5	28.8	81.8	24.3	84.6	27.9	80.7	23.0	81.6	24.6	85.5	29.3	79.9	22.4
B. vinsonii Winnie	80.9	23.4	85.1	28.3	81.4	23.9	84.1	27.5	80.5	22.7	81.7	24.4	85.3	28.6	79.9	22.4
B. vinsonii CIP103738	80.7	23.5	85.1	28.4	81.4	24.0	84.3	27.6	80.45	23.0	81.6	24.5	85.4	29.0	79.8	22.4
B. washoeensis	81.7	24.3	84.6	27.9	82.1	24.8	84.3	27.6	81.1	23.6	82.6	25.4	85.7	28.8	80.3	22.9

Column A and B represented ANI values and dDDH values, respectivel
