# Supplementary material for: Genomic characterization of eight novel Bartonella species from bats and ectoparasites reveals phylogenetic diversity and host adaptation
Source: PLoS Negl Trop Dis. 2025 Oct 23;19(10):e0013646. doi: 10.1371/journal.pntd.0013646 (PMC12574864; doi:10.1371/journal.pntd.0013646)
Supplement: S2 Table — (PDF) [file pntd.0013646.s003.pdf]

S2 Table. Core gene, accessory gene, unique gene, and pseudogene counts statistics on pan-genome annotation of the eight bat-borne novel *Bartonella* spp. of this study.

Strains	Core Genes	Shell Genes	Cloud Genes	Pseudogenes
B10	176	1,907	1,343	82
B35	186	1,988	1,190	83
B41	120	2,161	1,279	68
B17	210	1,845	1,542	102
B30	144	2,115	1,402	79
B23	331	2,468	2,398	113
B39	372	1,986	2,556	151
B12	320	1,759	2,080	89

Core genes are shared by all genomes and are involved in essential biological functions. Shell genes are present in some genomes and contribute to species diversity. Cloud genes occur in only a few genomes, often acquired via horizontal gene transfer, and may confer unique phenotypic traits or adaptations.

S2 Table. Statistics of COG functional annotation categories for the Core Genes of the eight bat-borne novel *Bartonella* spp.

COG categories	B10	B12	B17	B23	B30	B35	B39	B41
A (RNA processing and modification)	0	1	1	1	1	0	0	0

B (Chromatin structure and dynamics)	0	0	0	0	0	0	0	0
C (Energy production and conversion)	22	32	30	34	20	24	37	14
D (Cell cycle control, cell division, chromosome partitioning)	2	4	2	4	2	2	6	2
E (Amino acid transport and metabolism)	8	18	12	26	7	6	28	4
F (Nucleotide transport and metabolism)	13	23	12	20	9	15	24	7
G (Carbohydrate transport and metabolism)	7	14	5	12	3	5	14	1
H (Coenzyme transport and metabolism)	4	9	7	9	4	4	12	3
I (Lipid transport and metabolism)	11	16	14	21	8	11	21	8
J (Translation, ribosomal structure and biogenesis)	38	67	56	66	41	47	70	33
K (Transcription)	19	33	17	28	16	17	27	16
L (Replication, recombination and repair)	8	19	7	20	3	10	19	4
M (Cell wall/membrane/envelope biogenesis)	4	8	4	6	2	2	10	2
N (Cell motility)	1	0	0	1	0	2	1	0
O (Posttranslational modification, protein turnover, chaperones)	13	16	15	22	11	9	19	10
P (Inorganic ion transport and metabolism)	6	20	10	22	5	5	23	4
Q (Secondary metabolites biosynthesis, transport and catabolism)	2	3	3	4	2	0	3	0
R (General function prediction only)	1	1	1	1	1	0	0	0
S (Function unknown)	11	24	16	24	11	13	29	4
T (Signal transduction mechanisms)	10	14	10	14	6	6	13	5
U (Intracellular trafficking, secretion, and vesicular transport)	7	9	2	12	2	8	14	3

V (Defense mechanisms)	1	2	1	1	0	1	2	0
W (Extracellular structures)	0	0	0	0	0	0	0	0
X (Mobilome: prophages, transposons)	0	0	0	0	0	0	0	0
Y (Nuclear structure)	1	1	1	1	1	0	0	0
Z (Cytoskeleton)	0	0	0	0	0	0	0	0
