# Supplementary material for: Genomic characterization of eight novel Bartonella species from bats and ectoparasites reveals phylogenetic diversity and host adaptation
Source: PLoS Negl Trop Dis. 2025 Oct 23;19(10):e0013646. doi: 10.1371/journal.pntd.0013646 (PMC12574864; doi:10.1371/journal.pntd.0013646)
Supplement: S3 Table — (PDF) [file pntd.0013646.s004.pdf]

S3 Table. The information on the *Bartonella* strains used in this study.

Bartonella	Strains	NCBI Accession number	GC percent	Genome length (Mb)	Completeness (%)
B. alsatica	IBS 382T	GCF_902728125.1	37.0	1.7	99.5
B. ancashensis	20.00	GCF_001281405.1	38.5	1.5	99.5
B. australis	AUST/NH1	GCF_000341355.1	42.0	1.6	99.9
B. bacilliformis	KC583	GCF_000015445.1	38.0	1.4	95.2
B. birtlesii	IBS 325	GCF_000273375.1	37.5	1.8	99.4
B. bovis	91-4	GCF_000384965.1	37.5	1.6	99.8
B. callosciuri	DSM 28538	GCF_014203215.1	38.5	1.7	99.6
B. capreoli	DSM 21569	GCF_902813205.1	38.0	1.8	99.3
B. chomelii	DSM 21431	GCF_014138465.1	37.5	1.6	99.7
B. clarridgeiae	73	GCF_000253015.1	35.5	1.5	97.1
B. doshiae	NCTC 12862	GCF_000526895.1	38.0	1.8	97.7
B. elizabethae	NCTC 12898	GCF_900638615.1	38.5	2.0	97.7
B. florencae	R4	GCF_000312525.1	38.5	2.1	98.1
B. fuyuanensis	DSM 100694	GCF_014197255.1	36.5	1.9	98.4
B. gabonensis	669	GCF_903679515.1	38.0	2.0	99.7
B. grahamii	as4aup	GCF_000022725.1	38.0	2.4	99.6
B. harrusi	117A	GCF_024297065.1	38.5	2.3	96.1
B. henselae	Houston-I	GCF_002735245.1	38.5	2.0	99.8
B. koehlerae	C-29	GCF_000706625.1	37.5	1.7	99.7

B. kosoyi	Tel Aviv	GCF_003606325.2	38.5	2.3	98.8
B. krasnovii	OE 1-1	GCF_003606345.3	38.0	2.2	99.8
B. machadoae	46A	GCF_022559585.1	39.0	2.7	98.6
B. massiliensis	OS09	GCF_902150025.1	38.0	2.3	97.9
B. mastomydis	008	GCF_900185775.1	38.5	2.0	99.6
B. melophagi	K-2C	GCF_000278255.1	37.0	1.6	99.6
B. phoceensis	CIP107707	GCF_902825145.1	38.5	1.8	99.8
B. quintana	Toulouse	GCF_000046685.1	39.0	1.6	98.2
B. raoultii	094	GCF_019659805.1	37.0	2.0	99.6
B. rattaustraliani	AUST/NH4	GCF_000312565.1	39.0	2.2	98.6
B. rattimassiliensis	15908	GCF_000278215.1	36.5	2.2	98.9
B. refiksaydamii	RSKK 19006	GCF_902652675.1	38.5	1.9	98.0
B. rochalimae	BMGH	GCF_000706645.1	35.5	1.5	98.2
B. schoenbuchensis	CCUG 50783	GCF_902810545.1	37.5	1.6	99.8
B. senegalensis	OS02	GCF_000312545.1	38.5	2.0	96.3
B. sp.	1-1C	GCF_002810325.1	36.0	1.6	98.1
B. sp.	11B	GCF_002022625.1	36.0	1.6	97.7
B. sp.	114	GCF_002022645.1	36.0	1.6	93.6
B. sp.	A1379B	GCF_002022485.1	36.0	1.5	97.1
B. sp.	CDC_skunk	GCF_002022545.1	36.0	1.6	97.9
B. sp.	Coyote22sub2	GCF_002022565.1	36.0	1.6	97.4

B. sp.	DB5-6	GCF_000278115.1	38.5	2.1	99.3
B. sp.	JB15	GCF_002022605.1	35.5	1.5	91.9
B. sp.	JB63	GCF_002022665.1	35.5	1.5	95.7
B. sp.	Raccoon60	GCF_002022585.1	36.0	1.6	95.7
B. sp.	WD12.1	GCF_002022415.1	38.0	1.8	93.5
B. sp.	WD16.2	GCF_002022505.1	37.5	1.8	92.7
B. tamiae	Th307	GCF_000279995.1	38.0	2.3	96.1
B. taylorii	8TBB	GCF_000278295.1	38.4	2.0	99.4
B. tribocorum	CIP 105476	GCF_000196435.1	39.0	2.6	99.3
B. vinsonii	NCTC12905	GCF_900638635.1	39.0	2.0	99.3
B. vinsonii	ATCC 700727	GCF_902825115.1	38.5	1.8	99.5
B. vinsonii	Winnie	GCF_000341385.1	39.0	1.8	98.9
B. vinsonii	CIP 103738	GCF_902825235.1	39.0	1.9	99.0
B. washoeensis	085-0475	GCF_000278195.1	39.0	2.0	99.9

S3 Table. 16S rRNA gene sequences used for phylogenetic analyses in this study.

Bartonella	Accession number
Bartonella alsatica IBS 382	NR_025272.1
Bartonella ancashensis 20.00	NR_137367.1
Bartonella australis AUST/NH1	NR_115816.1
Bartonella bacilliformis KC583	NR_044743.1
Bartonella bovis 91-4	NR_025121.1
Bartonella capreoli IBS 193	NR_025120.1
Bartonella clarridgeiae Houston-2	NR_036961.1
Bartonella doshiae R18	NR_029368.1
Bartonella elizabethae F9251	NR_025889.1
Bartonella grahamii V2	NR_029366.1
Bartonella henselae Houston-1	NR_074335.2
Bartonella massiliensis OS09	NR_178467.1
Candidatus *Bartonella mayotimonensis*	FJ376733.1
Bartonella queenslandensis AUST/NH12	NR_116176.1
Bartonella quintana S13	HQ014621.1
Candidatus *Bartonella raoultii* 094	KF792121.1
Bartonella rattimassiliensis 15908	NR_115255.1
Bartonella rochalimae BMGH	NR_115858.1
Bartonella sp. H-Nig-956	MN504662.1
Bartonella sp. KK182	MN252307.1
Bartonella sp. M1-44	MN251854.1
Bartonella sp. SK157	MN252312.1
Bartonella tamiae Th339	EF672729.1
Bartonella taylorii M6	NR_029367.1
Bartonella tribocorum IBS 506	NR_025278.1
Bartonella vinsonii subsp. *berkhoffii* 93-C01	NR_029153.1
Bartonella washoensis	AF070463.1
Brucella abortus 544	NR_042460.1
Bartonella sp. B10	PX237371
Bartonella sp. B12	PX237372
Bartonella sp. B17	PX237373
Bartonella sp. B23	PX237374
Bartonella sp. B30	PX237375

Bartonella sp. B35	PX237376
Bartonella sp. B39	PX237377
Bartonella sp. B41	PX237378

S3 Table. *ftsZ* gene sequences used for phylogenetic analyses in this study.

Bartonella	NCBI Accession number
Bartonella alsatica	AF467763.1
Bartonella birtlesii	AF467762.1
Bartonella bovis I724598	KR733181.1
Bartonella capreoli Honshu-18.2	AB703115.1
Bartonella clarridgeiae M9HN-SHQ	EU571942.1
Bartonella doshiae	AF467754.1
Bartonella elizabethae	AF467760.1
Bartonella grahamii Chengde-Rn34	OP382395.1
Bartonella kosoyi Jize-Aa47	OP382425.1
Candidatus *Bartonella mayotimonensis*	FJ376734.1
Bartonella queenslandensis AUST/NH11	EU111779.1
Bartonella quintana H56SC	KY436621.1
Bartonella rattimassiliensis Chengde-Rn25	OP382394.1
Bartonella rochalimae SM318006	DQ676490.1
Bartonella sp. H-Nig-956	MN529319.1
Bartonella sp. KK182	KY232170.1
Bartonella sp. strain M1-44	MF288090.1
Bartonella sp. SK157	KY232157.1
Bartonella tamiae Th339	EF605282.1
Bartonella taylorii Chengde-Rn40	OP382396.1
Bartonella tribocorum Jize-Aa49	OP382426.1
Bartonella vinsonii subsp. *berkhoffii*	AF467764.1
Bartonella washoensis ER14-3	AB519080.1
Brucella abortus	NZ_KB850274.1
Bartonella sp. B10	PX229487
Bartonella sp. B12	PX229488
Bartonella sp. B17	PX229489
Bartonella sp. B23	PX229490
Bartonella sp. B30	PX229491

Bartonella sp. B35	PX229492
Bartonella sp. B39	PX229493
Bartonella sp. B41	PX229494

S3 Table. *gltA* gene sequences used for phylogenetic analyses in this study.

Bartonella	NCBI Accession number
Bartonella alsatica 14SMX	MT821835.1
Bartonella ancashensis CO20	KC178618.1
Bartonella australis Aust/NH1	DQ538395.1
Bartonella bacilliformis Bb-22	OR515780.1
Bartonella birtlesii	AF204272.1
Bartonella bovis CMTUV	OR061369.1
Bartonella capreoli B28980	HM167503.1
Bartonella elizabethae INR20-01	MZ802851.1
Bartonella grahamii B12511	AB426656.1
Bartonella henselae BAR94	PQ227838.1
Bartonella kosoyi Jize-Aa47	OP382453.1
Candidatus *Bartonella mayotimonensis*	FJ376732.1
Bartonella phoceensis K054P	PQ279859.1
Bartonella queenslandensis O014P	PQ279871.1
Bartonella quintana Iran_10	PV057184.1
Bartonella rochalimae 02	PP466317.1
Bartonella sp. H-Nig-956	MN529467.1
Bartonella sp. KK182	KY232212.1
Bartonella sp. M1-44	HM545139.1
Bartonella sp. SK157	KY232199.1
Bartonella tamiae Th339	EF605280.1
Bartonella taylorii LL117	OQ808860.1
Bartonella tribocorum P033S	PQ279889.1
Bartonella vinsonii AlgPI3	OR826328.1
Bartonella washoeensis CZ-22	MZ089836.1
Brucella abortus	NZ_KB850274.1
Bartonella sp. B10	PX243279
Bartonella sp. B12	PX243280
Bartonella sp. B17	PX243281

Bartonella sp. B23	PX243282
Bartonella sp. B30	PX243283
Bartonella sp. B35	PX243284
Bartonella sp. B39	PX243285
Bartonella sp. B41	PX243286

S3 Table. *rpoB* gene sequences used for phylogenetic analyses in this study.

Bartonella	NCBI Accession number
Bartonella alsatica	AF165987.1
Bartonella ancashensis CO20	KC178619.1
Bartonella bacilliformis Bb-22	OR515799.1
Bartonella birtlesii	AB196425.1
Bartonella bovis ST33	OP894361.1
Bartonella callosciuri	AB529929.1
Bartonella capreoli B28980	HM167505.1
Bartonella clarridgeiae	AF165990.1
Bartonella doshiae	AF165991.1
Bartonella elizabethae THSKR-032	JX158366.1
Bartonella florencae R4	HM622143.1
Bartonella grahamii KR28	JN647927.1
Bartonella henselae TaDr273	MT411872.1
Bartonella koehlerae 149E	OP948204.1
Candidatus *Bartonella mayotimonensis*	FJ376736.1
Bartonella phoceensis GS164	OR994030.1
Bartonella queenslandensis AUST/NH11	EU111790.1
Bartonella quintana MF1-1	LC031778.1
Candidatus *Bartonella raoultii* 094	MZ934401.1
Bartonella rochalimae BR2	FJ147196.1
Bartonella sp. H-Nig-956	MN529418.1
Bartonella sp. KK182	KY232299.1
Bartonella sp. M1-44	MF288101.1
Bartonella sp. SK157	KY232286.1
Bartonella tamiae Th307	EF605285.1
Bartonella taylorii	AF165995.1
Bartonella tribocorum 2015-193	MH547334.1

Bartonella vinsonii subsp. *berkhofii*	AF165989.1
Bartonella washoensis 08S-0475	AB674244.1
Brucella abortus	NZ_KB850274.1
Bartonella sp. B10	PX243287
Bartonella sp. B12	PX243288
Bartonella sp. B17	PX243289
Bartonella sp. B23	PX243290
Bartonella sp. B30	PX243291
Bartonella sp. B35	PX243292
Bartonella sp. B39	PX243293
Bartonella sp. B41	PX243294
