# Supplementary material for: Genomic characterization of eight novel Bartonella species from bats and ectoparasites reveals phylogenetic diversity and host adaptation
Source: PLoS Negl Trop Dis. 2025 Oct 23;19(10):e0013646. doi: 10.1371/journal.pntd.0013646 (PMC12574864; doi:10.1371/journal.pntd.0013646)
Supplement: S4 Table — (PDF) [file pntd.0013646.s005.pdf]

S4 Table. Positive selection analysis of core genes in eight bat-borne novel *Bartonella* spp. was conducted.

Bartonella Strains	Preferred name
B10	*nuoD*
	nuoI
	lldD
	pnp
	dnaA
	trwG
	phyR
	gltK
	purC
B12	*ptsH*
B17	*cyoB*
	Icd
B30	*himD*
	accD
	rplT
	fur1
B35	*recR*
	atpE
	nuoI
	lolD
	QU41_16170
	livF
	rpsH
	prs
	asd
	mdh
B39	*atpB*
	rpoZ
	lon
	glpX
	rpsC
	gst

B41	*nusA*
