# Supplementary material for: Genomic characterization of eight novel Bartonella species from bats and ectoparasites reveals phylogenetic diversity and host adaptation
Source: PLoS Negl Trop Dis. 2025 Oct 23;19(10):e0013646. doi: 10.1371/journal.pntd.0013646 (PMC12574864; doi:10.1371/journal.pntd.0013646)
Supplement: S5 Table — (PDF) [file pntd.0013646.s006.pdf]

S5 Table. Functional Categories of COG Protein Function Annotations.

COG category	Description
A	RNA processing and modification
B	Chromatin structure and dynamics
C	Energy production and conversion
D	Cell cycle control, cell division, chromosome partitioning
E	Amino acid transport and metabolism
F	Nucleotide transport and metabolism
G	Carbohydrate transport and metabolism
H	Coenzyme transport and metabolism
I	Lipid transport and metabolism
J	Translation, ribosomal structure and biogenesis
K	Transcription
L	Replication, recombination and repair
M	Cell wall/membrane/envelope biogenesis
N	Cell motility
O	Posttranslational modification, protein turnover, chaperones
P	Inorganic ion transport and metabolism
Q	Secondary metabolites biosynthesis, transport and catabolism
R	General function prediction only
S	Function unknown
T	Signal transduction mechanisms
U	Intracellular trafficking, secretion, and vesicular transport
V	Defense mechanisms
W	Extracellular structures
X	Mobilome: prophages, transposons
Y	Nuclear structure
Z	Cytoskeleton

S5 Table. Software, versions, and parameter settings used in this study

Software	Version	Parameters
IQ-TREE	2.4.0	-m MFP -bb 1000
Fastp	1.0.1	Default
NanoFilt	2.3.0	-l 500 --headcrop 50 --tailcrop 50
Spades	4.1.0	Default
BUSCO	5.7.1	Default
CheckM	1.2.3	Default

Prokka	1.14.6	Default
Mafft	7.525	Default
FastANI	1.34	Default
Diamond	2.1.13	Default
Roary	3.13.0	Default
Gffread	0.12.7	Default
PAL2NAL	14.1	Default
HyPhy	2.5.71	Default
Python	2.7	Default
Bowtie2	2.5.4	-p 8 -D 30 -L 10
FreeBayes	1.3.9	Default
snpEff	5.2	Default
