# Supplementary material for: Genomic characterization of eight novel Bartonella species from bats and ectoparasites reveals phylogenetic diversity and host adaptation
Source: PLoS Negl Trop Dis. 2025 Oct 23;19(10):e0013646. doi: 10.1371/journal.pntd.0013646 (PMC12574864; doi:10.1371/journal.pntd.0013646)
Supplement: S6 Table — (PDF) [file pntd.0013646.s007.pdf]

S6 Table. Primers used for PCR in this study

Target species	Geng	Primers	Sequences (5′-3′)	PCR products (bp)	Reference
Bartonella	16S rRNA	16S-F	AGGGCTYAACCCTGG	1526	[1]
		16s-R	TACCCTGTTACYRCTC		
	gltA	gltA-F	GCTATGTTTGCRTTBTATAA	751	[2]
		glA-R	GATCMTCAATYATYKCTTYCCM		
	rpoB	rpoB-F	CGCATTGGCGTACAYCCTACR	852	[2]
		rpoB-R	GTAGTCCDATTGAAACDCCD		
	ftsZ	ftsZ-F	ATTAATCDGGRGCRGCCAGA	885	[2]
		ftsZ-R	ACRGAYGCDCRGATRAYACY		
Bat	*cytB*	cytB-F	CCATGAGGCCAAATATCCTTCTGAGG	604	[3]
		cytB-R	TTGGCCAATGATAATGTAKGGRTGTTC		
Bat fly	*COI*	LCO1490	GGTCAACAAATCATAAAGATATTGG	658	[4]
		HCO2198	TAAACTTCAGGGTGACCAAAAAATCA		
Bat mite	*COI*	C1-J-2183	CAACATTTATTTTGATTTTTTGG	658	[5]
		C1-J-2797	GGATAATCTGAATAACGTCGAGG		

References

1. Evans NJ, Brown JM, Demirkan I, Singh P, Getty B, Timofte D, et al. Association of unique, isolated treponemes with bovine digital dermatitis lesions. J Clin Microbiol. 2009;47(3):689-96. Epub 20090114. doi: 10.1128/jcm.01914-08. PubMed PMID: 19144804; PubMed Central PMCID: PMCPMC2650952.
2. Bai Y, Hayman DT, McKee CD, Kosoy MY. Classification of *Bartonella* strains associated with straw-colored fruit bats (*Eidolon helvum*) across Africa using a multi-locus sequence typing platform. PLoS Negl Trop Dis. 2015;9(1):e0003478. Epub 20150130. doi: 10.1371/journal.pntd.0003478. PubMed PMID: 25635826; PubMed Central PMCID: PMCPMC4311972.
3. Li ZM, Xiao X, Zhou CM, Liu JX, Gu XL, Fang LZ, et al. Human-pathogenic relapsing fever *Borrelia* found in bats from Central China phylogenetically clustered together with relapsing fever borreliae reported in the New World. PLoS Negl Trop Dis. 2021;15(3):e0009113. Epub 20210318. doi: 10.1371/journal.pntd.0009113. PubMed PMID: 33735240; PubMed Central PMCID: PMCPMC7971464.
4. Folmer O, Black M, Hoeh W, Lutz R, Vrijenhoek R. DNA primers for amplification of mitochondrial cytochrome c oxidase subunit I from diverse metazoan invertebrates. Mol Mar Biol Biotechnol. 1994;3(5):294-9. PubMed PMID: 7881515.
5. Bruyndonckx N, Dubey S, Ruedi M, Christe P. Molecular cophylogenetic relationships between European bats and their ectoparasitic mites (Acari, Spinturnicidae). Mol Phylogenet Evol. 2009;51(2):227-37. Epub 20090221. doi: 10.1016/j.ympev.2009.02.005. PubMed PMID: 19236931.
